# Comparison of clinical outcomes with proximal femoral nail anti-rotation versus dynamic hip screw for unstable intertrochanteric femoral fractures: A meta-analysis

**DOI:** 10.1097/MD.0000000000032920

**Published:** 2023-02-10

**Authors:** Cong Zhang, Zhangxin Chen, Mengyuan Wang, Wei Chen, Zhenqi Ding

**Affiliations:** a Department of Orthopedics, The 909th Hospital, School of Medicine, Xiamen University, Zhangzhou, China; b School of Medicine, Xiamen University, Xiamen, China.

**Keywords:** dynamic hip screw, meta-analysis, proximal femoral nail anti-rotation, unstable femoral intertrochanteric fractures

## Abstract

The aim of this meta-analysis was to evaluate the advantages and disadvantages of proximal femoral nail anti-rotation (PFNA) versus dynamic hip screw (DHS) for the treatment of unstable intertrochanteric fractures, including the available evidence drawn from the literature.

**Methods:**

A systematic search was conducted to identify available and relevant randomized controlled trials and retrospective comparative observational studies regarding PFNA compared against DHS in treating unstable femoral intertrochanteric fractures in Embase, PubMed, Cochrane Library, Web of Science, and Scopus Online up to February 12, 2022. Data from the included studies were extracted independently by 2 reviewers and analyzed using RevMan 5.3, and the quality of the studies was assessed.

**Results:**

Five randomized controlled trials and 12 observational studies were recruited and met the inclusion criteria, which consisted of 1332 patients with PFNA and 1271 patients with DHS. The results of the meta-analysis showed that, compared with the DHS, PFNA exhibited a beneficial role in postoperative Harris Hip Scores, operation time, intraoperative blood loss, length of hospital stay, fracture healing time and full weight-bearing time, limb shortening, cutout, reoperation, union problems, the varus collapse of the femoral head/neck, and infection; however, DHS was superior to PFNA in hidden blood loss (relative risk [RR] = 139.81, 95% confidence interval [CI] [136.18, 143.43], *P* < .00001), postoperation drainage (RR = −17.85, 95% CI [−30.10, −5.60], *P* = .004), total blood loss (RR = 50.34, 95% CI [42.99, 57.69], *P* < .00001), and femoral shaft fracture (RR = 4.72, 95% CI [1.15, 19.32], *P* = .03) treated by DHS were significantly decreased, compared with those by PFNA; however, no significant differences were observed in tip–apex distance, fixation failures, screw migration, or other complicants between the 2 surgical methods.

**Conclusion:**

Analysis of a large number of relevant clinical indicators available shows that PFNA has better clinical manifestation than DHS in treating unstable femoral intertrochanteric fractures.

## 1. Introduction

With the aging population, intertrochanteric fractures have increased over the years.^[1]^ Surgery is the primary treatment, including extramedullary and intramedullary fixation. Both fixations could have an excellent clinical outcome for treating stable intertrochanteric.^[2]^ However, it remains controversial for optimal fixations in treating unstable intertrochanteric fractures.

Dynamic hip screw (DHS) is a typical extramedullary fixation with a locking-type pressure device. It has been the gold standard for treating intertrochanteric fractures because of its good clinical results, low nonunion rate, and minor fixation failures.^[3]^ However, multiple literatures have shown that screw cutout, internal rotation of the femoral shaft, and collapse of the femoral head would correlate with DHS.^[4–6]^ Proximal femoral nail anti-rotation (PFNA) is a special helical blade intramedullary device which compacts cancellous bone by sliding compression, thus providing better axial compressive forces and rotational stability.^[7]^ Compared with DHS, PFNA has minor invasion, a lower degree of soft tissue injury, a lower reoperation rate, a shorter recovery time, and better functional recovery.^[8,9]^ However, PFNA remains a series of problems, such as screw cutout, postoperative thigh pain, femoral shaft fractures, high requirements for blood transfusion, high hidden blood loss, and fat embolism.^[10,11]^

To date, however, data comparing clinical outcomes of PFNA and InterTAN nail are lacking. The present meta-analysis aimed to comprehensively compare the clinical outcomes of 2 techniques and determine the advantages and disadvantages of the 2 internal fixation methods.

## 2. Methods

### 2.1. Search strategy

**Figure 1.**
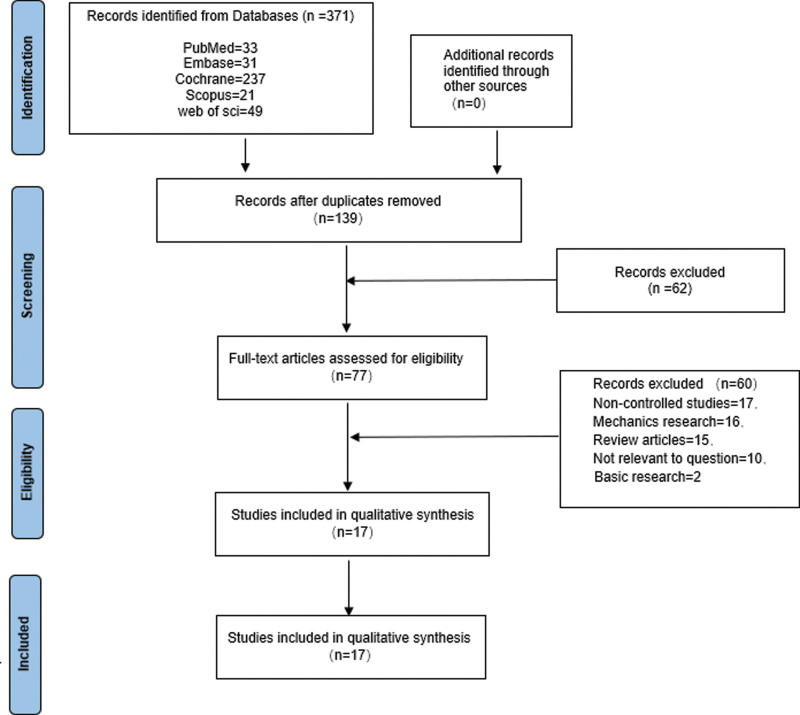
Flow diagram of literature selection.

This systematic review and meta-analysis is reported following the Preferred Reporting Items for Systematic Reviews and Meta-Analyses Statement and was registered at the International Prospective Register of Systematic Reviews (number CRD42022310412). Relevant articles published before February 2022 were searched in the following databases according to the Cochrane Collaboration’s search strategy: PubMed, EMBASE, Cochrane Central Register of Controlled Trials, Web of Science, and Scopus; the reference lists of relevant literature were further filtered to ensure the comprehensiveness and variety of the review. We used the following search terms: (“‘PFNA’” or “‘proximal femoral nail anti-rotation’”) and (“‘DHS’” or “‘dynamic hip screw’”) and (“‘unstable intertrochanteric fractures’” or “‘unstable extracapsular hip fractures’” or “‘unstable trochanteric fractures’” or “‘unstable pertrochanteric fractures’”). A flow chart provides an overview of the details of the selection process (Fig. 1).

### 2.2. Inclusion and exclusion criteria

Two authors independently screened all titles and abstracts using the PICO (P: Patients; I: Intervention; C: Comparison; O: Outcomes) framework in the following manner: population: individuals with unstable intertrochanteric fractures; intervention: PFNA; comparator: DHS; outcomes: operation time, blood loss, length of hospital stay, time to union, tip–apex distance (TAD), Harris Hip Score (HHS), cutout, implant failure, screw migration, reoperation, infection, union problems, femoral shaft fractures, femoral head abnormalities, and other complications; and study design: a number of studies (prospective, randomized controlled trials (RCTs) or comparative observational studies published in any language) were eligible to be included in the review. This study included clinical trials with at least one of the primary clinical outcomes described above, although some studies had a follow-up period of fewer than 12 months. However, review articles, biomechanical research, animal trials, uncontrolled experiments, and duplicate or multiple publications from the same study were excluded. All differences and discrepancies were resolved through discussion and consensus among the reviewers, and, if necessary, the corresponding authors were consulted.

### 2.3. Data extraction

In eligible studies, relevant data were extracted independently by both authors using pre-defined data extraction sheets and then cross-checked; additionally, any discrepancies were resolved by consensus between reviewers. The primary data sources were the primary study authors, time of publication, sample size, patient baseline characteristics (e.g., age and gender), patient fracture type, duration of follow-up, and reported outcomes, which were summarized in Table 1.

**Table 1:** The characteristics of included studies.

Study, yr	PFNA/DHS				Follow-up/mo	Type of study
	Sample size	Age (yr)	Gender (male/female)	Fracture type (number)		
Andalib, 2020^[12]^	38/55	61.45 ± 17.0 64.40 ± 15.5	43/50	A*	12	RCT
Zehir, 2015^[13]^	96/102	77.22 ± 6.82 76.86 ± 6.74	76/122	A2.1 49/A2.2 87/A2.3 62	6	RCT
Huang, 2015^[7]^	30/30	75.07 ± 7.87 74.01 ± 7.25	32/28	Evans†III 26/IV 23/V 11	12–24	RCT
Sinan, 2014^[13]^	96/102	77.22 6.82 76.86 6.74	76/122	A2.1 49/A2.2 87/A2.3 62	6	RCT
Aktselis, 2014^[14]^	36/35	82.9 ± 5.8 83.1 ± 6.5	15/56	A2.2-A2.3	12	RCT
Chua, 2013^[15]^	25/38	75 77	31/32	A2 50/A3 11	12	RS
Li, 2013^[16]^	42/50	72.8 ± 5.8 72.5 ± 5.3	49/43	EvansII34/IIB 38/III 14/R 6	10–18	RS
Wang, 2018^[17]^	90/68	74 ± 14 74 ± 11	57/101	EvansIIA 85/IIB63/III10	6–18	RS
Lu, 2019^[18]^	45/38	88.35 ± 6.12 88.77 ± 5.52	44/39	EvansIIIA 39/IIIB33/IV11	12	RS
Müller, 2020^[19]^	200/75	82.6 ± 8.00 82.6 ± 8.00	42/233	A2	24	RS
Tahir, 2018^[20]^	32/30	71.6610.59 72.13 ± 9.06	29/33	A2.2-A2.3	12	RS
Zhang, 2012^[21]^	216/168	75.4/73.4	181/201	A2 212/A3 99	Unclear	RS
Yu, 2016^[22]^	186/177	78.3/77.7	153/210	A2 104/A3 101	37–42	RS
Huang, 2012^[23]^	43/72	72.8 ± 4.9 72.5 ± 5.5	32/83	A2 69	6–36	RS

Data are presented as n or mean ± standard deviation.

DHS = dynamic hip screw, PFNA = proximal femoral nail anti-rotation, RCT = randomized controlled trial, RS = retrospective study.

* Arbeitsge-meinschaft für Osteosynthesefragen/Orthopaedic Trauma Association. † Evans-Jensen classification.

### 2.4. Quality assessment and risk of bias

Methodological quality was assessed independently by 2 authors according to the Cochrane Handbook 5.1.0, for Systematic Reviews of Interventions. Seven items were required for inclusion: selection bias, random sequence generation; selection bias, assignation concealment; performance bias, blind to study participants and study staff; detection bias, blinding of outcome evaluation; reporting bias, selective reporting; attrition bias, outcome data incompleteness; and other biases. In our review, we measured the full methodological quality of each study as “yes” (low risk of bias), “unclear” (unclear risk of bias), or “no” (high risk of bias) and used it to get the risk of bias plot and summary of the bias through Review Manager version 5.30 (RevMan, the Cochrane Collaboration, Oxford, UK). All divergent views were resolved through team consensus.

### 2.5. Statistical analysis

Review Manager software version 5.3 (Copenhagen: The Nordic Cochrane Centre, the Cochrane Collaboration, 2014) was used to perform the meta-analysis. The relative risk (RR) was used for evaluating dichotomous outcomes such as cutout, implant failure, screw migration, reoperation, infection, union problems, femoral shaft fractures, femoral head abnormalities, and other complications. In the case of continuous data and recording the mean difference (MD) (RR) with a 95% confidence interval (CI), a *P* value <.05 was considered statistically significant. Studies were tested for heterogeneity using *P* and *I*^2^ statistics; in addition, if heterogeneity between studies was not significant (*I*^2^ < 50% and *P* > .1), a fixed-effects model was used; otherwise, a random-effects model (50% ≤ *I*^2^ ≤ 100%)was chosen. Also, subgroup analyses were performed according to the different types of studies included. In the absence of significant heterogeneity across studies, a combined analysis was used to use the available data best. In addition, sensitivity analysis was performed by excluding 1 study in each iteration to verify the robustness of the study results.

### 2.6. Ethical statement

The data analyzed in this study were taken from previously published studies and therefore did not need ethical approval.

## 3. Results

### 3.1. Study selection and characteristics of the selected studies

Initially, 371 articles were identified as potentially relevant, and 136 remained after the duplicates were removed, 62 of which had been excluded by preliminary screening; the full text was carefully screened to exclude 77 articles, leaving 17 for detailed evaluation. Between January 2010 and February 2022, 17 studies (n = 2603 patients) met the inclusion and exclusion criteria, including 5 RCTs and 12 observational studies. A total of 2603 patients were relatively equally distributed between the PFNA group (n = 1332 patients) and the DHS group (n = 1271 patients), and all studies were followed up for at least 6 months. Given that no blinding was reported in the included studies, all RCTs were classified as having an unclear risk of bias. Most observational studies were judged to be of sufficient quality according to the Grading of Recommendations Assessment, Development and Evaluation checklist.

### 3.2. Quality assessment and risk of bias

**Figure 2.**
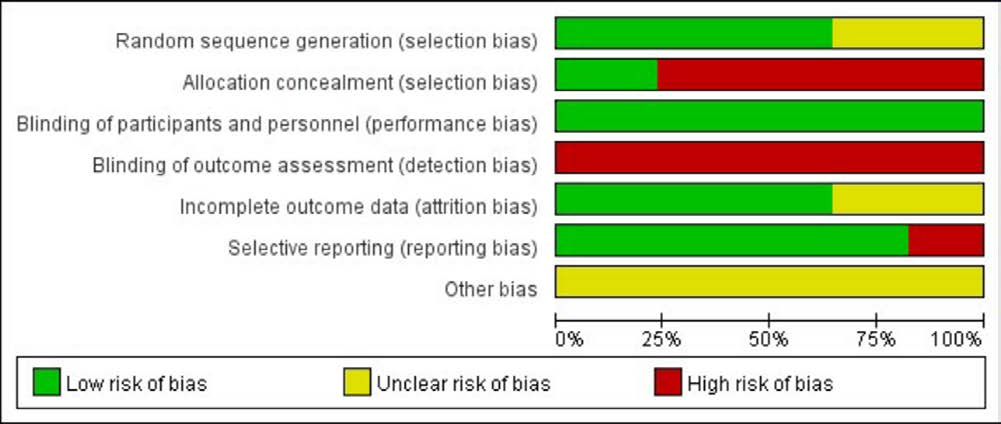
The risk of bias summary of the included studies. (+ represents yes; − represents no; ? represents not clear).

**Figure 3.**
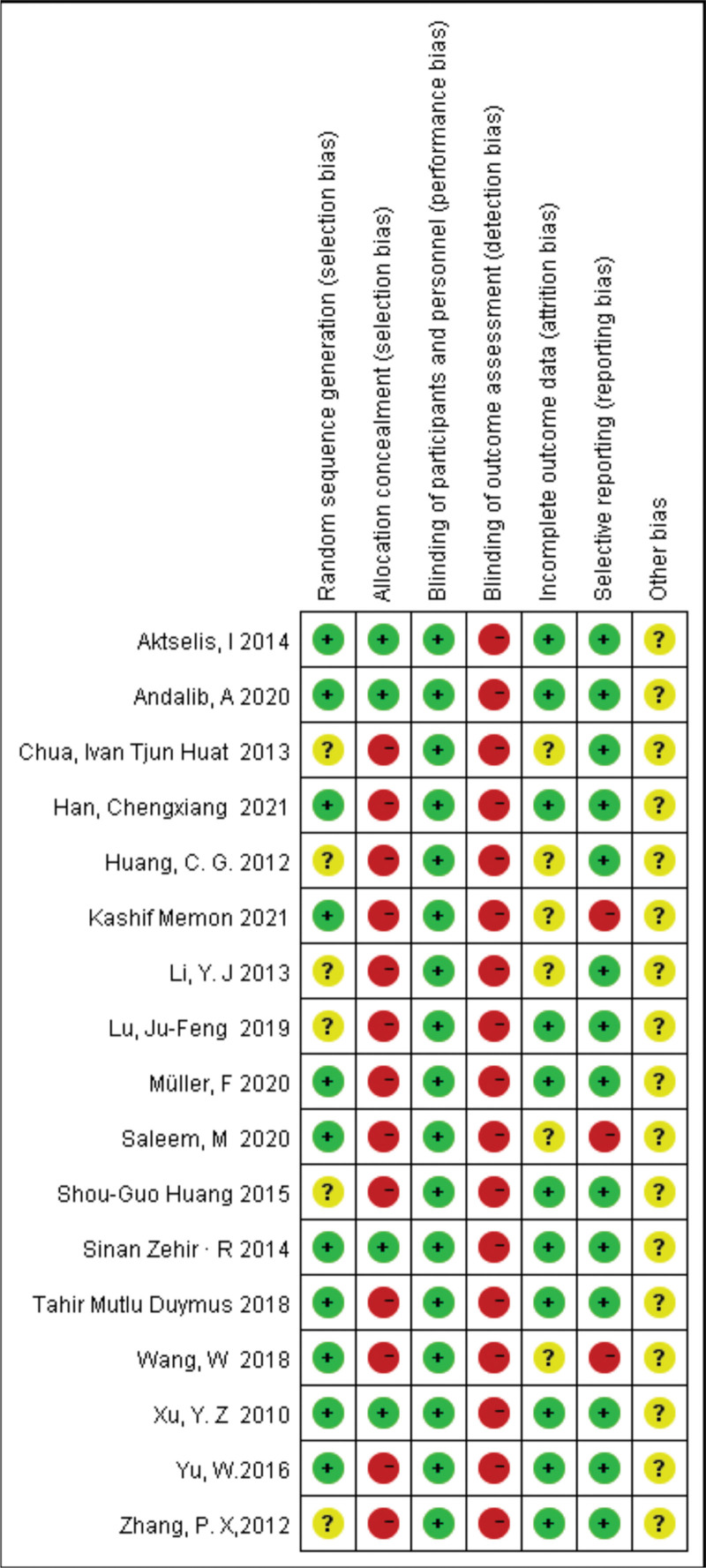
The risk of bias graph of the included studies.

The methodological quality of all included RCTs and observational studies was assessed according to the Cochrane Handbook for Systematic Reviews of Interventions. Detailed risk of bias estimates for eligible studies are presented in Figures 2 and 3.

### 3.3. Clinical outcomes

#### 3.3.1. Primary outcome.

**Figure 4.**
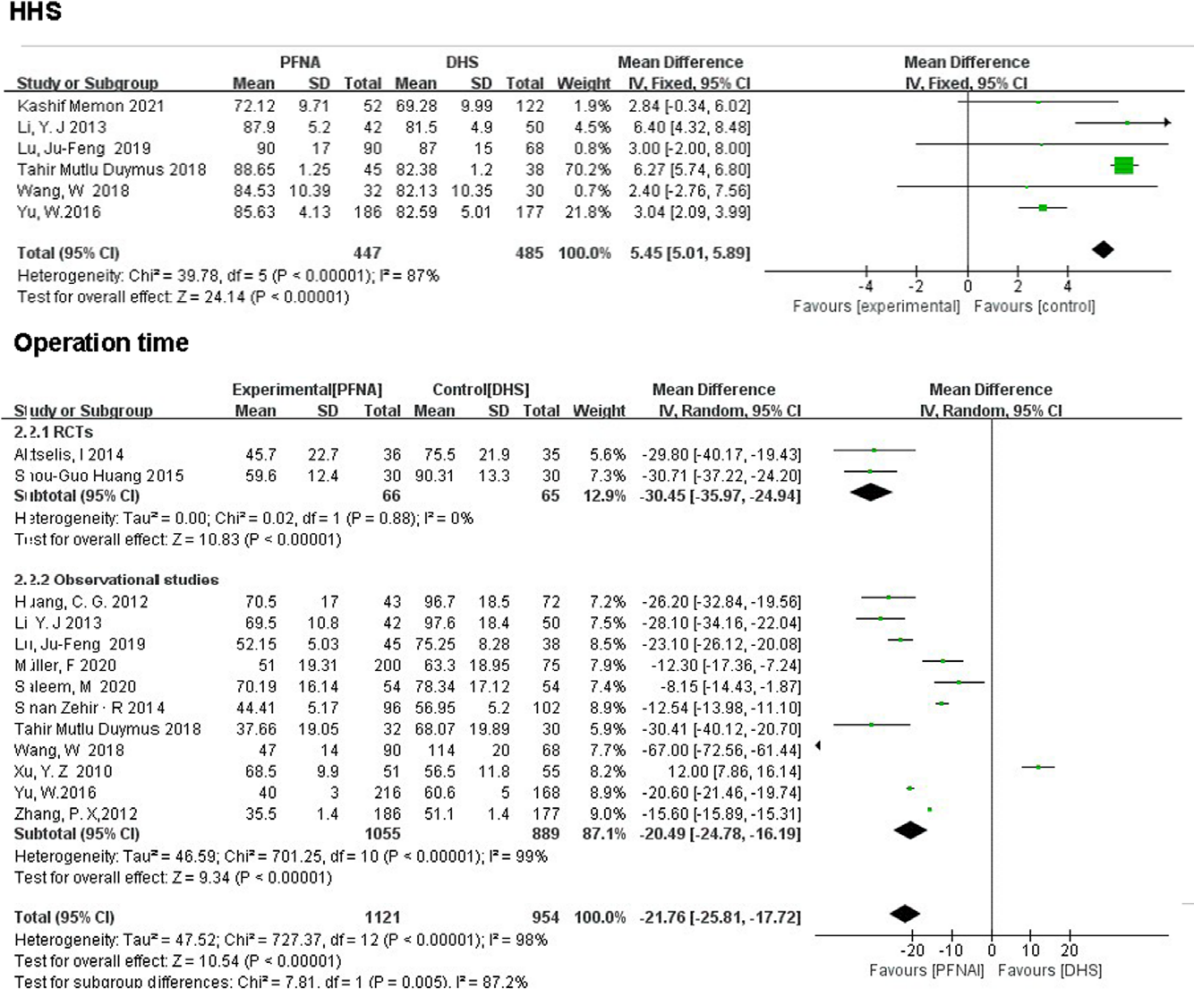
A forest plot diagram showed HHS, operation time. HHS = Harris Hip Score.

HHS was recorded in all 6 studies (n = 932). There were significant differences between PFNA and DHS (MD = 5.45, 95% CI [5.01, 5.89], *P* < .00001; Fig. 4). using the random-effects model as a result of moderate heterogeneity (*χ*^2^ = 39.78, df = 5, *P* < .00001, *I*² = 87%).

#### 3.3.2. Secondary outcomes.

##### 3.3.2.1. Operation time.

Operation time was reported in 13 studies (n = 2075 patients). Significant differences were observed in PFNA versus DHS in both RCTs (MD = −30.45, 95% CI [−35.97, −24.94], *P* < .00001; Fig. 4) and observational studies (MD = −20.49, 95% CI [−24.78, −16.19], *P* < .00001; Fig. 4) using the random-effects model with the statistical heterogeneity (RCTs: *χ*^2^ = 0.02, df = 1, *P* = .88, *I*² = 0%; observational studies: *χ*^2^ = 701.25, df = 10, *P* < .00001, *I*^2^ = 99%).

##### 3.3.2.2. Intraoperative blood loss.

**Figure 5.**
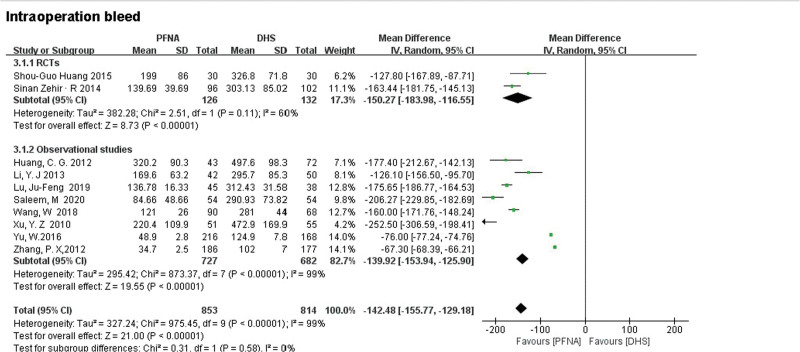
A forest plot diagram showed intraoperative blood loss.

Seven studies consisting of 2 RCTs and 5 observational studies showed the outcome of intraoperative blood loss. The RCTs (MD = −150.27, 95% CI [−183.98, −116.55], *P* < .00001; Fig. 5) and observational studies (MD = −139.92, 95% CI [−153.94, −125.90], *P* < .00001; Fig. 5) all showed PFNA had less intraoperative bleeding than DHS. Heterogeneity was tested using a random-effects model(RCTs: *χ*^2^ = 2.51, df = 1, *P* = .11, *I*^2^ = 60%; observational studies: *χ*^2^ = 873.37, df = 7, *P* < .00001, *I*^2^ = 99%).

##### 3.3.2.3. Hidden blood loss.

**Figure 6.**
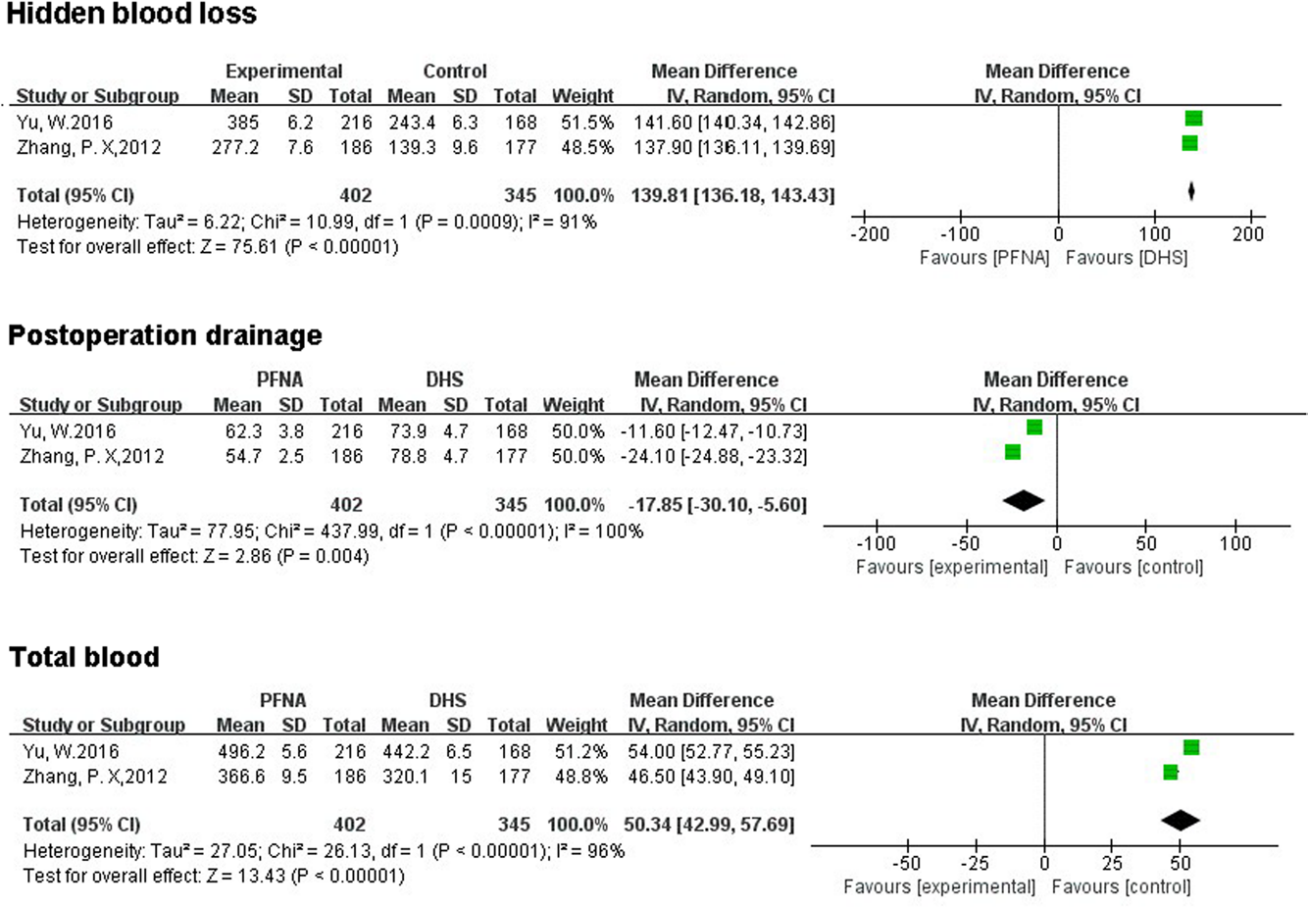
A forest plot diagram showed hidden blood loss, postoperation drainage, and total blood loss.

A total of 747 patients were included in 2 studies that reported latent blood loss. There was a statistically significant difference between the 2 groups (RR = 139.81, 95% CI [136.18, 143.43], *P* < .00001]; Fig. 6). Random effects model was used to test heterogeneity, (*χ*^2^ = 10.99, df = 1, *P* = .0009, *I*^2^ = 91%).

##### 3.3.2.4. Postoperation drainage.

A total of 747 patients were included in 2 literatures. There was a significant statistical difference between the 2 groups (MD = −17.85, 95% CI [−30.10, −5.60], *P* < .00001]; Fig. 6). Random effects model was used to test heterogeneity, (*χ*^2^ = 437.99, df = 1, *P* < .00001, *I*^2^ = 100%).

##### 3.3.2.5. Total blood loss.

Total postoperative blood loss was reported in 2 literatures, involving a total of 747 patients. There was a significant statistical difference between the 2 groups (MD = 50.34, 95% CI [42.99, 57.69], *P* < .00001]; Fig. 6). Random effects model was used to test heterogeneity, (*χ*^2^ = 26.13, df = 1, *P* < .00001, *I*^2^ = 96%).

##### 3.3.2.6. TAD.

**Figure 7.**
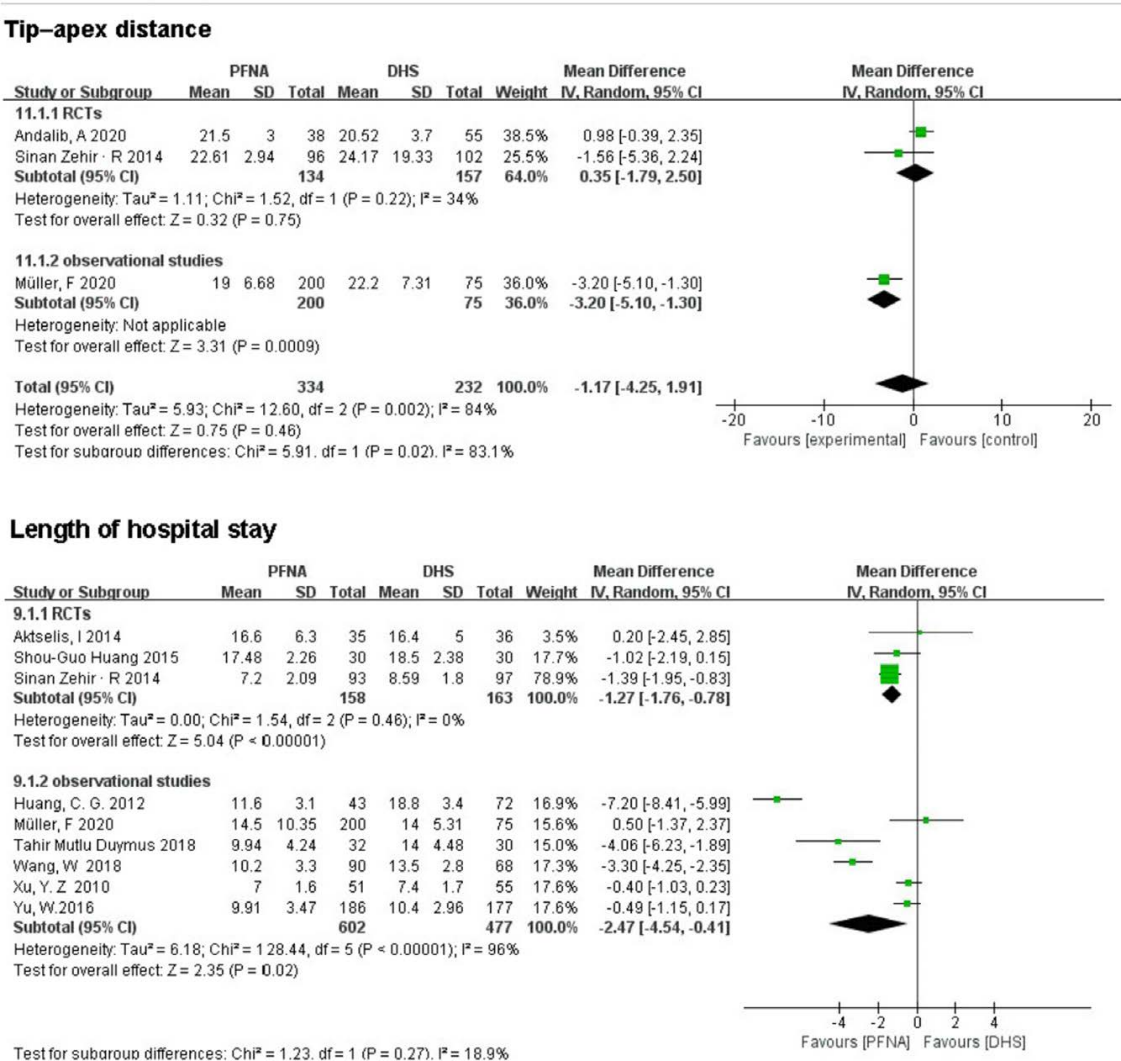
A forest plot diagram showed tip–apex distance, and length of hospital stay.

TAD was reported in 2 randomized controlled studies and 1 observational study analysis involving 566 patients. A observational study showed statistically significant differences between the 2 groups (MD = 3.20, 95% CI [−5.10, −1.30], *P* = .0009; Fig. 7). RCTs showed no significant difference between the 2 groups (MD = 0.35, 95% CI [−1.79, 2.50], *P* = .75; Fig. 7). Combined analysis showed no significant difference in TAD between PFNA and DHS. Heterogeneity was tested using a random-effects model (RCTs: *χ*^2^ = 2.51, df = 1, *P* = .11, *I*^2^ = 60%; observational study: *χ*^2^ = 29.73, df = 4, *P* < .00001, *I*^2^ = 87%).

##### 3.3.2.7. Length of hospital stay.

A total of 9 works of literature reported the length of hospital stay, including 1400 patients. There were statistically significant differences between the PFNA group and the DHS group in both RCTs and observational studies (RCTs: RR = −1.27, 95% CI [−1.76, −0.78], *P* < .00001; observational studies: RR = −2.47, 95% CI [−4.54, −0.41], *P* = .02; Fig. 7). Heterogeneity was tested using a random-effects model, and the retrospective subgroup showed high heterogeneity (*χ*^2^ = 34.56, df = 3, *P* < .00001, *I*^2^ = 91%), which may be related to the different health status among the groups.

##### 3.3.2.8. Time to union.

**Figure 8.**
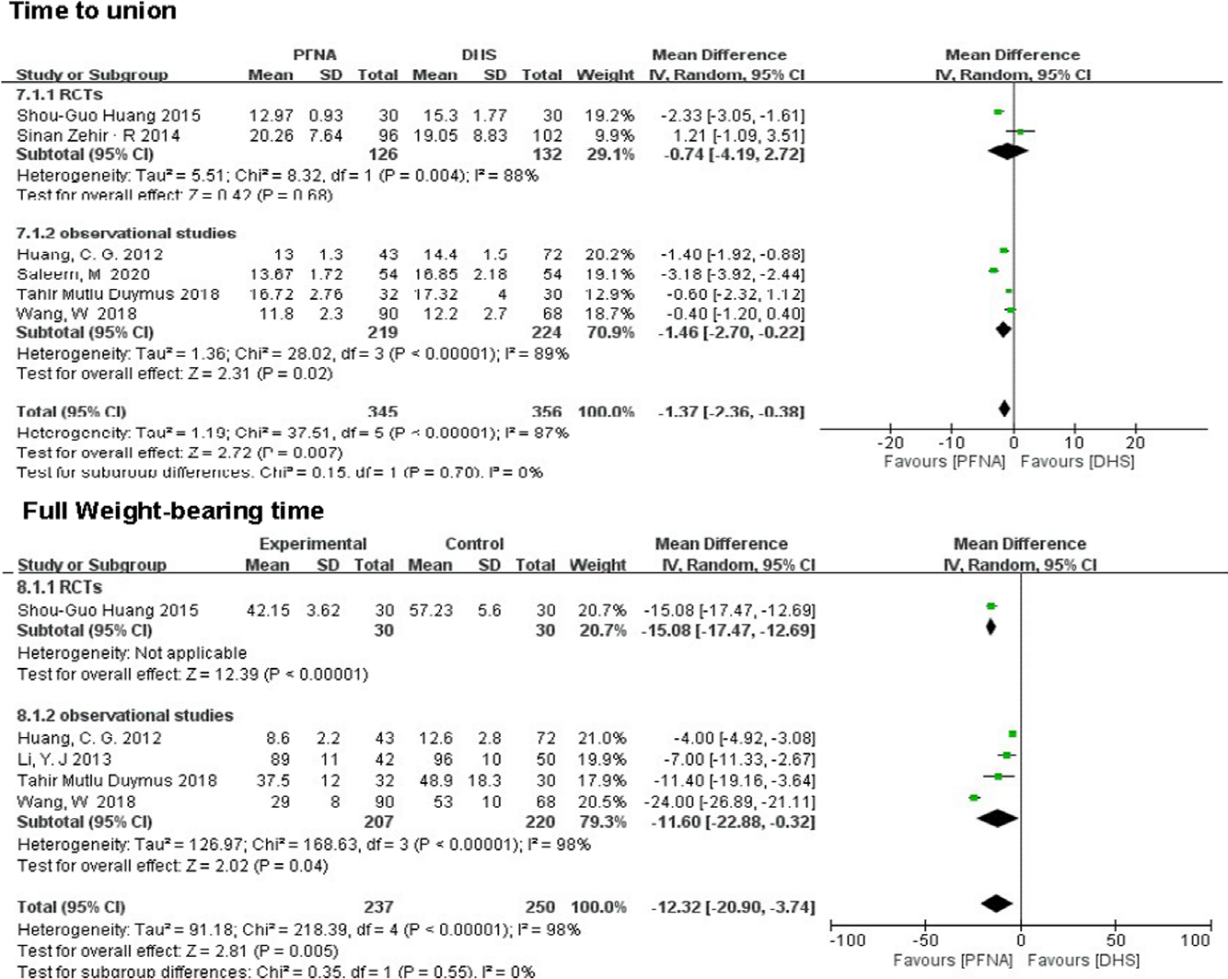
A forest plot diagram showed the time to union and full weight-bearing time.

A total of 6 literatures reported time to union, including 701 patients. In the RCTs, there was no significant difference between the PFNA group and the DHS group (MD = −0.74, 95% CI [−4.19, 2.72], *P* = .68; Fig. 8), but in the observational studies, Significant difference between the 2 groups (MD = 1.46, 95% CI (2.70, 0.22), *P* = .02; Fig. 8). After association analysis, the difference between the 2 groups remained significant (RR = −1.37, 95% CI [−2.36, −0.38], *P* = .007). Heterogeneity was tested using a random-effects model(RCTs: *χ*^2^ = 8.32, df = 1, *P* = .004, *I*^2^ = 88%; observational studies *χ*^2^ = 28.02, df = 3, *P* < .00001, *I*^2^ = 89%).

##### 3.3.2.9. Full weight-bearing time.

A total of 487 patients with early postoperative weight-bearing time were included in 5 literatures. RCTs (MD = −15.08, 95% CI [−17.47, −12.69], *P* < .00001; Fig. 8) and observational studies (MD = −11.60, 95% CI [−22.88, −0.32], *P* = .04; Fig. 8) showed a significant statistical difference between the 2 groups. Heterogeneity was tested using a random-effects model (*χ*^2^ = 218.39, df = 4, *P* < .00001, *I*^2^ = 98%).

##### 3.3.2.10. Limb shortening.

**Figure 9.**
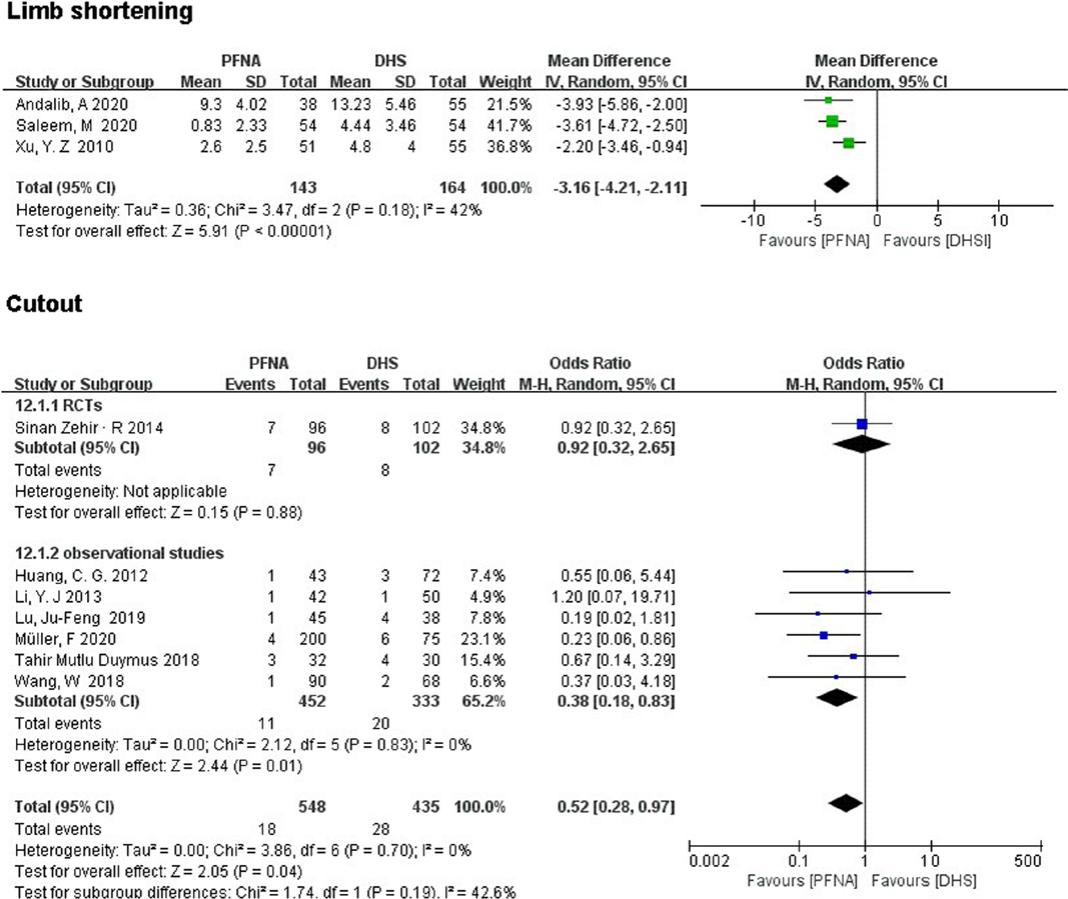
A forest plot diagram showed limb shortening and cutout.

A total of 3 observational studies (n = 307) reported limb shortening. Significant differences were found between the PFNA and DHS groups through subgroup analysis (RR = −3.16 [95% CI (−4.21, −2.11)], *P* < .00001; Fig. 9). Random effects model was used to test heterogeneity (*χ*^2^ = 3.47, df = 2, *P* = .18, *I*^2^ = 42%).

##### 3.3.2.11. Cutout.

A total of 983 patients with internal fixation were reported in 7 articles. Although no significant difference was found between PFNA and DHS in the RCTS (RR = 0.92, 95% CI [0.32, 2.65], *P* = .88; Fig. 9), However, a significant difference was found between the 2 groups in the observational studies (RR = 0.38, 95% CI [0.18, 0.83], *P* = .01; Fig. 9). Since no significant heterogeneity was found, fixed-effect models were used (*χ*^2^ = 3.86, df = 6, *P* = .70, *I*^2^ = 0%). Combined subgroup analysis based on low heterogeneity between the 2 subgroups showed no statistically significant difference between the 2 groups (RR = 0.52, 95% CI [0.28, 0.97], *P* = .04; Fig. 9).

##### 3.3.2.12. Internal fixation loosening.

**Figure 10.**
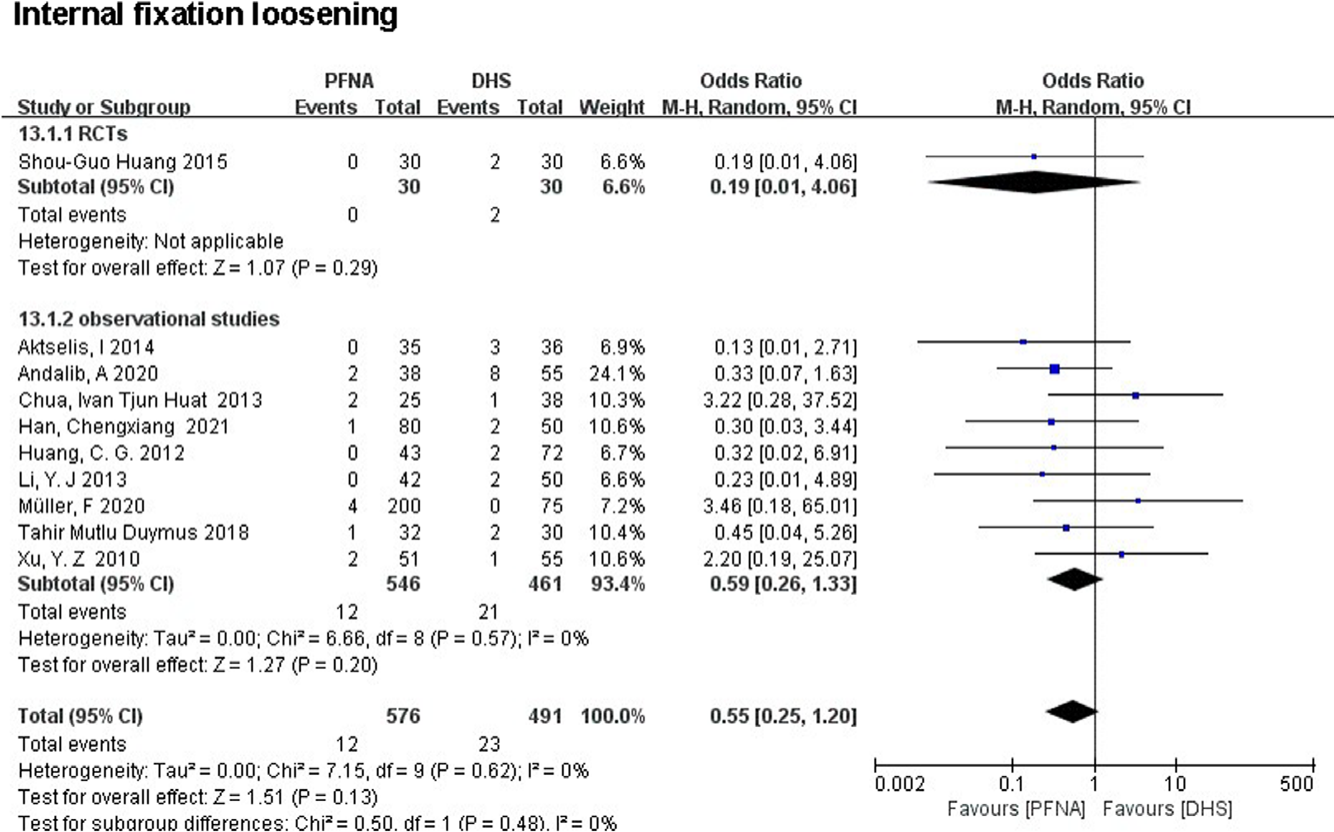
A forest plot diagram showed internal fixation loosening.

Internal fixation loosening includes internal fixation fracture, displacement, or retraction. A total of 10 literatures (n = 1067) reported the failure of internal fixation. There was no significant difference between the 2 groups in both RCTs (RR = 0.19, 95% CI [0.01, 4.06], *P* = .29; Fig. 10) and observational studies (RR = 0.59, 95% CI [0.26, 1.33], *P* = .20). Heterogeneity was tested using a fixed-effect model and a retrospective controlled study subgroup (RCTs: *χ*^2^ = 6.66, df = 8, *P* = .57; observational studies: *χ*^2^ = 7.15, df = 9 (*P* = .62); *I*^2^ = 0%).

##### 3.3.2.13. Screw migration.

**Figure 11.**
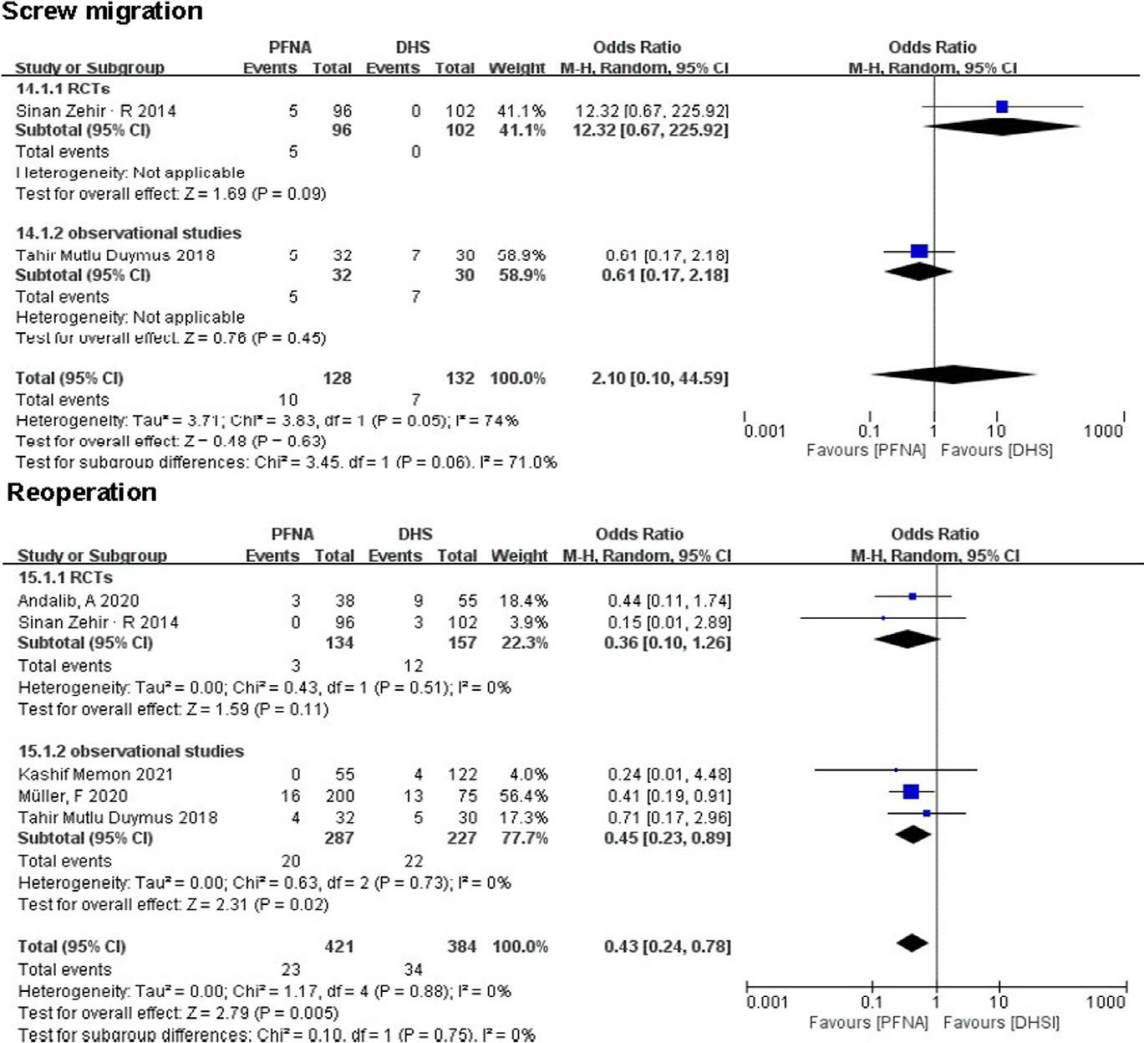
A forest plot diagram showed screw migration and reoperation.

A total of 2 literatures (n = 260) reported screw displacement. No significant difference was found between PFNA and DHS in both RCTs and observational studies through subgroup analysis (RCTs: RR = 12.32, 95% CI [0.67, 225.92], *P* = .09; observational studies: RR = 2.10, 95% CI [0.10, 44.59], *P* = .45; Fig. 11). Since no significant heterogeneity was found, the pooled effects were calculated using a fixed effect model (total: *χ*^2^ = 3.83, df = 1, *P* = .05, *I*^2^ = 74%).

##### 3.3.2.14. Reoperation.

A total of 805 patients were reported for reoperation in 5 literatures. RCTs (RR = 0.36, 95% CI [0.10, 1.26], *P* = .51; Fig. 11) and observational studies (RR = 0.45, 95% CI [0.23, 0.89], *P* = .73; Fig. 11) showed no significant difference in reoperation between PFNA group and DHS group. Heterogeneity was tested using a fixed-effect model (RCTs: *χ*^2^ = 0.43, df = 1; *I*^2^ = 0%; observational studies: *χ*^2^ = 0.63, df = 2; *I*^2^ = 0%). Due to the low heterogeneity of the 2 groups, a combined analysis of the 2 subgroups showed a lower rate of reoperation in the PFNA group than in the DHS group (RR = 0.43, 95% CI [0.24, 0.78], *P* = .005; Fig. 11).

##### 3.3.2.15. Union problems.

**Figure 12.**
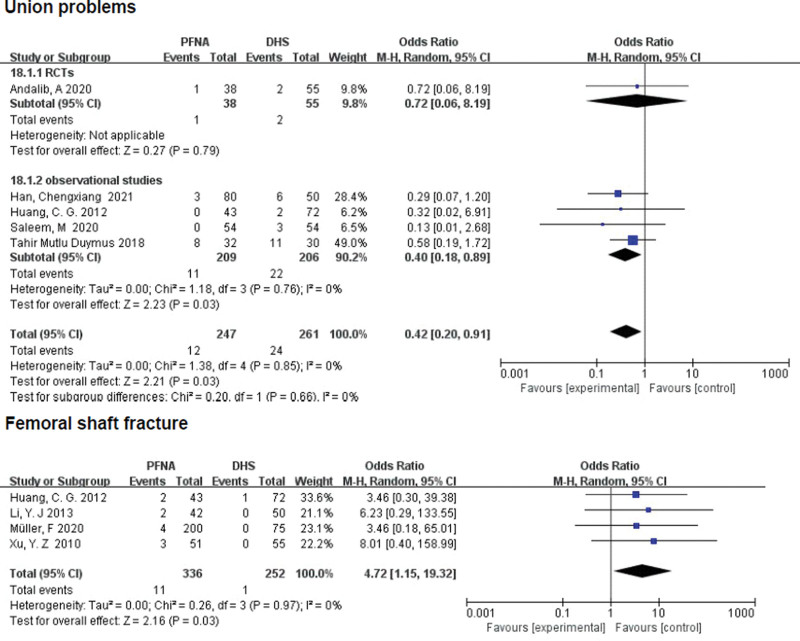
A forest plot diagram showed union problems and femoral shaft fracture.

A total of 5 literatures (n = 508) reported fracture union problems, including delayed union, malunion and nonunion. In a single RCTS, there was no significant difference between the PFNA group and the DHS group (RR = 0.72, 95% CI [0.06, 8.19], *P* = .79; Fig. 12), while observational studies showed that significant difference was found between the 2 groups (RR = 0.40, 95% CI [0.18, 0.89], *P* = .03; Fig. 12). Heterogeneity was tested using a fixed-effect model (*χ*^2^ = 1.38, df = 4, *P* = .85, *I*^2^ = 0%). Combined subgroup analysis based on low heterogeneity between the 2 groups showed that significant difference was found between the 2 groups (RR = 0.42, 95% CI [0.20, 0.91], *P* = .03).

##### 3.3.2.16. Femoral shaft fracture.

Results of femoral shaft fractures were reported in 4 articles (n = 588). In the 4 RCTs, PFNA showed no significant difference in postoperative femoral shaft fracture compared with DHS (RR = 4.72, 95% CI [1.15, 19.32], *P* = .03; Fig. 12). Heterogeneity was tested using a fixed-effect model (*χ*^2^ = 0.26, df = 3, *P* = .97; *I*^2^ = 0%).

##### 3.3.2.17. Hip varus.

**Figure 13.**
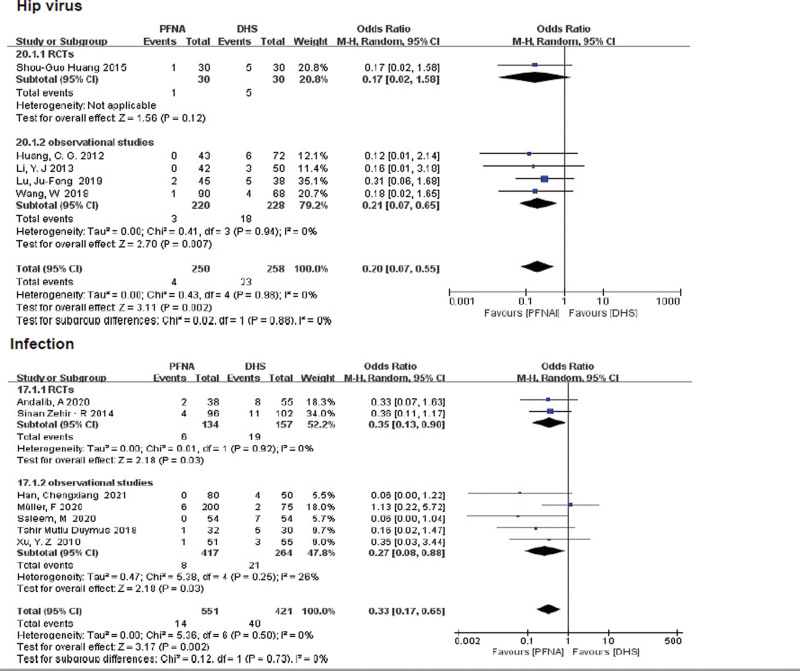
A forest plot diagram showed hip varus and infection.

A total of 508 patients with hip varus were included in 5 literatures. One RCT study showed no significant difference between PFNA and DHS (RR = 0.17, 95% CI [0.02, 1.58], *P* = .12), while there was a significant difference between the 2 groups in observational studies (RR = 0.21, 95% CI [0.07, 0.65], *P* = .007; Fig. 13). No significant heterogeneity was found between the 2 groups by fixed-effect model (*χ*^2^ = 0.43, df = 4, *P* = .98, *I*^2^ = 0%). Based on the low heterogeneity of the 2 subgroups, a combined subgroup analysis showed statistical differences between the 2 groups (RR = 0.20, 95% CI [0.07, 0.55], *P* = .002; Fig. 13).

##### 3.3.2.18. Infection.

Infection includes superficial wound infection and deep wound infection. A total of 972 patients with postoperative wound infection were reported in 7 literatures. Both RCTs and retrospective control subgroups showed a lower incidence of postoperative infection in PFNA than in DHS (RCTS: RR = 0.35, 95% CI [0.13, 0.90]), *P* = .03, Fig. 13; retrospective control subgroup: RR = 0.27, 95% CI = [0.08, 0.88], *P* = .03, Fig. 13). Heterogeneity was tested using a fixed-effect model (RCTs: *χ*^2^ = 0.01, df = 1, *P* = .92, *I*^2^ = 0%; observational studies *χ*^2^ = 5.38, df = 4, *P* = .25, *I*^2^ = 26%).

##### 3.3.2.19. Other complications.

**Figure 14.**
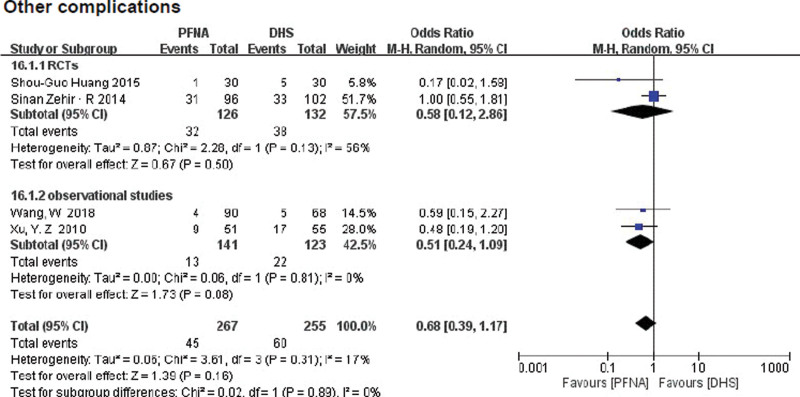
A forest plot diagram showed other complications.

Other complications include deep vein thrombosis, pulmonary embolism, decompensated heart failure, urinary tract infections, pneumonia, and pressure ulcers. These results were reported in 4 literatures (n = 522). Subgroup analysis of the randomized control group (RR = 0.58, 95% CI [0.12, 2.86], *P* = .50, Fig. 14) and the retrospective control group(RR = 0.51, 95% CI [0.24, 0.19], *P* = .81, Fig. 14) showed no significant difference between the PFNA group and the DHS group. Heterogeneity was tested using fixed-effect model (RCTs: *χ*^2^ = 2.28, df = 1, *P* = .13, *I*^2^ = 56%; observational studies: *χ*^2^ = 0.06, df = 1, *P* = .81, *I*^2^ = 0%).

### 3.4. Sensitivity analysis and publication bias

To test the stability and reliability of the present findings, We conducted a sensitivity analysis by iteratively excluding one study at a time to be showing similar heterogeneity before and after the removal of each study. To confirm the robustness of the findings. Results were reliable with the exception of HHS and full weight-bearing outcomes. In terms of HHS score, heterogeneity did not change significantly after excluding a study that caused heterogeneity^[23]^ (*χ*^2^ = 8.76, df = 4, *P* = .07, *I*^2^ = 54%, see Supplemental Figure S1, Supplemental Digital Content, http://links.lww.com/MD/I450, which demonstrates the comparison of results before and after deletion), which may be related to different physical qualities of patients. In addition, patients’ active degree of postoperative functional exercise may also affect the results. In terms of early weight-bearing, there was no significant change in the heterogeneity of other studies^[20]^ after excluding one study that caused heterogeneity (observational studies: *χ*^2^ = 5.09, df = 2, *P* = .08, *I*^2^ = 61%; total: *χ*^2^ = 74.74, df = 3, *P* < .00001, *I*^2^ = 96%, see Supplemental Figure S2, Supplemental Digital Content, http://links.lww.com/MD/I451, which demonstrates the Comparison of results before and after deletion), which may be due to the different surgical skills of the orthopedic surgeons included in the study.

## 4. Discussion

The number of patients with intertrochanteric fractures was increased.^[12,13]^ According to Arbeitsgemeinschaftfür Osteosynthesefragen fracture classification, A1 and A2-1 are stable intertrochanteric fractures, while A2-2, A2-3, and A3 are unstable intertrochanteric fractures.^[14]^ PFNA and DHS are the most common surgical methods for internal fixation of unstable intertrochanteric fractures, but which is more clinically advantageous is still controversial.^[15]^ Zhang^[16]^ found that, compared with DHS, PFNA had advantages such as less intraoperative blood loss, shorter fluoroscopy time, lower reoperation, and lower internal fixation failure rate through meta-analysis. However, due to the relatively low quality and credibility of evidence in the included literature, it could not be concluded that PFNA was superior to DHS. Shen^[17]^ compared the clinical efficacy of PFNA and DHS through meta-analysis, and the author did not recommend the use of DHS. However, only 5 articles with 534 patients were included, which reduced its statistical detection ability. Ma^[18]^ found that PFNA was recommended for unstable fractures through meta-analysis. However, this conclusion needs further demonstration due to the high risk of deviation and the lack of discussion on the relationship between fracture classification and clinical results. Parker^[19]^ found in a retrospective cohort study that intramedullary fixation had a lower incidence of postoperative complications and implant failure than extramedullary fixation. However, the authors only describe the statistical results without discussing the mechanism in depth. Given this, we remained skeptical of the relevant conclusions of this study and conducted a comparative analysis of the clinical efficacy of PFNA and DHS. A total of 1741 patients were included in this study, including 5 randomized controlled studies and 12 retrospective controlled studies, which enabled us to compare more clinical indicators. The latest comparison of clinical indicators between the 2 groups provides additional evidence for the selection of treatment options for unstable intertrochanteric fractures.

In terms of postoperative Harris, operation time, postoperative blood loss, hospital stay, early weight-bearing time, and limb shortening, current evidence is consistent with previous research results,^[18,20]^ indicating that PFNA is superior to DHS in these clinical outcomes. Contrary to previous results, our study found a statistically significant difference in fracture healing time between the 2 surgical procedures, supported by both RCT and observational subgroup analyses. It is not hard to understand that PFNA can allow patients to move to the ground earlier and perform hip function recovery training earlier.^[21]^ Early postoperative weight-bearing walking can provide appropriate axial stress stimulation to the fracture end, promoting fracture healing.^[22]^

Based on some of the other categorical variables listed here, the data suggest that DHS has a higher risk of postoperative wound infection, delayed healing, and nonunion. The current pooled data showed that DHS has a higher risk of postoperative wound infection, delayed healing, and nonunion, which may be related to the invasive surgical methods and mechanical properties of DHS. Consistent with these conclusions, Gadegone et al^[23]^ found that DHS can provide controllable compression force at the fracture end. However, DHS requires a more extensive surgical field, more destruction of soft tissue and blood supply, and anatomical reduction. Compared with PFNA implanted minimally invasive. All of these will increase the risk of postoperative infection, the varus collapse of the femoral head, delayed union, and nonunion. Meanwhile, Memon et al^[24]^ believed that the DHS should be in direct contact with the bone surface at both fracture ends. The periosteum beneath the steel plate must be removed further to destroy the blood supply to the affected part and affect fracture healing. In a randomized controlled trial comparing the 2 techniques, Reindl^[25]^ found a higher incidence of nonunion and malunion, due to the biomechanical properties of extramedullary devices that allow for direct and complete weight-bearing.

For unstable intertrochanteric fractures, axial flexion and extension stability and rotational stability provided by internal fixation after fracture reduction are critical. Weiser^[26]^ found that PFNA was centrally fixed through mechanical experiments. Compared with DHS, the force wall was shorter and closer to the loading line of lower limbs, thus reducing the stress bending degree at the top. Huang et al^[7]^ found that the PFNA spiral blade device directly hammered into the bone neck, reducing cancellous bone loss. Meanwhile, a large area of spiral blade contacted with bone, thus providing greater compression force, axial flexion, and rotation stability for the fracture end. In a randomized controlled study, Adeel^[27]^ found that the DHS was not effective in stabilizing unstable intertrochanteric fractures, especially comminuted fractures with combined posterior medial or greater trochanteric regions. Authors found that a fracture may cause this at the hip screw entry point or the bone fragment separation behind the screw. In a literature review,^[28]^ the authors found that after DHS operation, it is easy to occur lateral separation of the greater trochanteric fragments, displacement of small trochanter fracture pieces, medial instability, rotation or compression of femoral head and neck, and even hip varus. These may explain why PFNA is superior to DHS in hip varus, cut out, and reoperation, consistent with the current analysis.

In the current study, PFNA was compared with DHS in terms of intraoperative blood loss, hidden blood loss, postoperative drainage, and postoperative total blood loss. The results showed that PFNA had less intraoperative blood loss but was higher than DHS in terms of hidden blood loss, postoperative drainage, and postoperative total blood loss, which is consistent with previous studies.^[29,30]^ Hidden blood loss is often ignored in clinical practice, which tends to aggravate other complications or promote the occurrence of other complications. The cause and mechanism of latent blood loss are still unclear. Erskine et al suggested that internal bleeding may occur during the reaming step of intramedullary needle fixation.^[31]^ Mcmanus speculated that a large amount of blood entering the medullary cavity and muscle space during the perioperative period was the leading cause of invisible blood loss.^[32]^ For patients with low preoperative hemoglobin and renal insufficiency, attention should be paid to closely testing the basic conditions of patients and adjusting them to the best condition for surgery, as well as paying attention to closely testing patients’ vital signs during and after surgery, focusing on hemoglobin, hemorrhagic shock prevalence, and drainage volumes. In addition, in terms of postoperative complications, PFNA is prone to fracture of the femoral shaft around internal fixation due to its concentration of mechanical conduction stress. For postmenopausal women and patients with osteoporosis, attention should be paid to whether the internal fixation is loose due to the occurrence of periprosthetic fracture and regular postoperative review. We also compared and analyzed additional clinical outcomes, including postoperative TAD, internal fixation failure, screw displacement, and other complications. PFNA and DHS were similar in clinical outcomes, with no statistically significant differences.

The current study still has several inherent limitations. First, RCT studies were relatively few among the included studies, which may increase the potential bias. The current results would be more credible if more randomized controlled trials were included. Second, patients in the included literature had different follow-up times, which may lead to heterogeneity and bias. Despite some limitations of this study, the extracted data were of high quality according to inclusion and exclusion criteria.

## 5. Conclusions

Analysis of a large number of relevant clinical indicators available shows that PFNA has better clinical manifestation than DHS in treating unstable femoral intertrochanteric fractures.

## Acknowledgments

We would like to thank the subjects included in the current study and all physicians and nurses involved in the management of the patients.

## Author contributions

**Data curation:** Zhangxin Chen, Mengyuan Wang.

**Formal analysis:** Cong Zhang.

**Funding acquisition:** Zhenqi Ding.

**Project administration:** Zhenqi Ding.

**Software:** Cong Zhang, Mengyuan Wang.

**Supervision:** Mengyuan Wang.

**Writing – original draft:** Cong Zhang.

**Writing – review & editing:** Wei Chen, Zhenqi Ding.

## Supplementary Material




