# Supplementary material for: Comparison of clinical outcomes with proximal femoral nail anti-rotation versus dynamic hip screw for unstable intertrochanteric femoral fractures: A meta-analysis
Source: Medicine (Baltimore). 2023 Feb 10;102(6):e32920. doi: 10.1097/MD.0000000000032920 (PMC9907998; doi:10.1097/MD.0000000000032920)
Supplement: Supplementary file 1 [file medi-102-e32920-s001.pdf]

Fig.S1 Comparison of results before and after deletion

	Study or Subgroup	PFNA			DHS			Weight	Mean Difference
		Mean	SD	Total	Mean	SD	Total		IV, Fixed, 95% CI
☑	Kashif Memon 2021	72.12	9.71	52	69.28	9.99	122	1.9%	2.84 [-0.34, 6.02]
☑	Li, Y. J 2013	87.9	5.2	42	81.5	4.9	50	4.5%	6.40 [4.32, 8.48]
☑	Lu, Ju-Feng 2019	90	17	90	87	15	68	0.8%	3.00 [-2.00, 8.00]
☑	Tahir Mutlu Duymus 2018	88.65	1.25	45	82.38	1.2	38	70.2%	6.27 [5.74, 6.80]
☑	Wang, W 2018	84.53	10.39	32	82.13	10.35	30	0.7%	2.40 [-2.76, 7.56]
☑	Yu, W.2016	85.63	4.13	186	82.59	5.01	177	21.8%	3.04 [2.09, 3.99]
	Total (95% CI)			447			485	100.0%	5.45 [5.01, 5.89]
	Heterogeneity: Chi² = 39.78, df = 5 (P < 0.00001); I² = ...								
	Test for overall effect: Z = 24.14 (P < 0.00001)								

	Study or Subgroup	PFNA			DHS			Weight	Mean Difference
		Mean	SD	Total	Mean	SD	Total		IV, Fixed, 95% CI
☑	Kashif Memon 2021	72.12	9.71	52	69.28	9.99	122	6.5%	2.84 [-0.34, 6.02]
☑	Li, Y. J 2013	87.9	5.2	42	81.5	4.9	50	15.2%	6.40 [4.32, 8.48]
☑	Lu, Ju-Feng 2019	90	17	90	87	15	68	2.6%	3.00 [-2.00, 8.00]
☐	Tahir Mutlu Duymus 2018	88.65	1.25	45	82.38	1.2	38	0.0%	6.27 [5.74, 6.80]
☑	Wang, W 2018	84.53	10.39	32	82.13	10.35	30	2.5%	2.40 [-2.76, 7.56]
☑	Yu, W.2016	85.63	4.13	186	82.59	5.01	177	73.2%	3.04 [2.09, 3.99]
	Total (95% CI)			402			447	100.0%	3.52 [2.71, 4.33]
	Heterogeneity: Chi² = 8.76, df = 4 (P = 0.07); I² = 54%								
	Test for overall effect: Z = 8.52 (P < 0.00001)								
