# Supplementary material for: Comparison of clinical outcomes with proximal femoral nail anti-rotation versus dynamic hip screw for unstable intertrochanteric femoral fractures: A meta-analysis
Source: Medicine (Baltimore). 2023 Feb 10;102(6):e32920. doi: 10.1097/MD.0000000000032920 (PMC9907998; doi:10.1097/MD.0000000000032920)
Supplement: Supplementary file 2 [file medi-102-e32920-s002.pdf]

Fig.S2 Comparison of results before and after deletion

	Study or Subgroup	Experimental			Control			Weight	Mean Difference
		Mean	SD	Total	Mean	SD	Total		IV, Random, 95% CI
☑	8.1.1 RCTs								
☑	Shou-Guo Huang 2015	42.15	3.62	30	57.23	5.6	30	20.7%	-15.08 [-17.47, -12.69]
	Subtotal (95% CI)			30			30	20.7%	-15.08 [-17.47, -12.69]
	Heterogeneity: Not applicable								
	Test for overall effect: Z = 12.39 (P < 0.00001)								
☑	8.1.2 observational studies								
☑	Huang, C. G. 2012	8.6	2.2	43	12.6	2.8	72	21.0%	-4.00 [-4.92, -3.08]
☑	Li, Y. J 2013	89	11	42	96	10	50	19.9%	-7.00 [-11.33, -2.67]
☑	Tahir Mutlu Duymus 2018	37.5	12	32	48.9	18.3	30	17.9%	-11.40 [-19.16, -3.64]
☑	Wang, W 2018	29	8	90	53	10	68	20.5%	-24.00 [-26.89, -21.11]
	Subtotal (95% CI)			207			220	79.3%	-11.60 [-22.88, -0.32]
	Heterogeneity: Tau^2 = 126.97; Chi^2 = 168.63, df = 3 (P < 0.00001); I^2 = 98%								
	Test for overall effect: Z = 2.02 (P = 0.04)								
	Total (95% CI)			237			250	100.0%	-12.32 [-20.90, -3.74]
	Heterogeneity: Tau^2 = 91.18; Chi^2 = 218.39, df = 4 (P < 0.00001); I^2 = 98%								
	Test for overall effect: Z = 2.81 (P = 0.005)								
	Test for subgroup differences: Chi^2 = 0.35, df = 1 (P = 0.55), I^2 = 0%								

	Study or Subgroup	Experimental			Control			Weight	Mean Difference
		Mean	SD	Total	Mean	SD	Total		IV, Random, 95% CI
☑	8.1.1 RCTs								
☑	Shou-Guo Huang 2015	42.15	3.62	30	57.23	5.6	30	26.9%	-15.08 [-17.47, -12.69]
	Subtotal (95% CI)			30			30	26.9%	-15.08 [-17.47, -12.69]
	Heterogeneity: Not applicable								
	Test for overall effect: Z = 12.39 (P < 0.00001)								
☑	8.1.2 observational studies								
☑	Huang, C. G. 2012	8.6	2.2	43	12.6	2.8	72	27.6%	-4.00 [-4.92, -3.08]
☑	Li, Y. J 2013	89	11	42	96	10	50	25.0%	-7.00 [-11.33, -2.67]
☑	Tahir Mutlu Duymus 2018	37.5	12	32	48.9	18.3	30	20.5%	-11.40 [-19.16, -3.64]
☐	Wang, W 2018	29	8	90	53	10	68	0.0%	-24.00 [-26.89, -21.11]
	Subtotal (95% CI)			117			152	73.1%	-6.07 [-9.66, -2.48]
	Heterogeneity: Tau^2 = 6.03; Chi^2 = 5.09, df = 2 (P = 0.08); I^2 = 61%								
	Test for overall effect: Z = 3.31 (P = 0.0009)								
	Total (95% CI)			147			182	100.0%	-9.24 [-16.09, -2.39]
	Heterogeneity: Tau^2 = 43.96; Chi^2 = 74.74, df = 3 (P < 0.00001); I^2 = 96%								
	Test for overall effect: Z = 2.65 (P = 0.008)								
	Test for subgroup differences: Chi^2 = 16.77, df = 1 (P < 0.0001), I^2 = 94.0%								
